# Rhabdomyolysis during myelosuppression in a patient with central nervous system leukemia: A case report

**DOI:** 10.1097/MD.0000000000013091

**Published:** 2018-11-09

**Authors:** Ying Le, Huiqun Li, Chengyao Wan, Yuan Long, Zhenfang Liu

**Affiliations:** Hematology Department, First Affliated Hospital, Guangxi Medical University, Nanning, China.

**Keywords:** acute lymphoblastic leukemia, central nervous system leukemia, creatine kinase, rhabdomyolysis

## Abstract

**Rationale:**

Rhabdomyolysis is a potentially life-threatening syndrome and is a rare complication in patients with acute leukemia.

**Patient's concerns:**

A 20-year-old male was admitted to our hospital due to skin ecchymosis in his trunk and lower limbs for 10 days.

**Diagnoses:**

Based on the precise diagnosis of leukemia, namely cell morphology, immunology, cytogenetics, and molecular biological typing (MICM), the patient was diagnosed with acute T-lymphocytic leukemia (T-ALL).

**Interventions:**

The patient received hyper-Cyclophosphamide, Vincristine,Adriamycin, Dexamethasone (hyper-CVAD) regimen chemotherapy (methotrexate, pirarubicin, vincristine and dexamethasone alternating with methotrexate and cytarabine) for 3 courses of chemotherapy. After 3 months of treatment, the patient developed intermittent pain, blurred vision, and inarticulate speech. Therefore, the patient was considered as central nervous system leukemia (CNSL) and immediately received 2 courses of chemotherapy with hyper-CVAD-B combined with polyethylene glycol conjugated asparaginase (PEG-ASP).

**Outcomes:**

On the seventh day after the completion of chemotherapy, the patient was diagnosed with rhabdomyolysis because he complained of perianal pain and hematuria, and his creatine kinase (CK) increased suddenly to 3136 U/L. Finally, the patient died despite all kinds of active rescue.

**Lessons:**

Rhabdomyolysis may occur after chemotherapy of leukemia. When patients developed hematuria, muscle weakness, or even asymptomatic elevation of CK levels, physicians should pay attention to the occurrence of rhabdomyolysis and take active hydration treatment.

## 1 Introduction

Acute lymphoblastic leukemia (ALL) is a highly invasive and malignant disease of lymphoblastic cells, characterized by the clonogenic proliferation of abnormal T/B primitive lymphocyte in marrow, peripheral blood, and extramedullary tissue. ALL is the most common acute leukemia in children, and it also accounts for 20% of adult acute leukemia.^[1]^ Although a variety of chemotherapeutic drug developments have been used in the treatment of adult ALL in recent years, it has still lower long-term survival and higher recurrence rate compared with children. ALL often involves the central nervous system which indicates poor prognosis.

Rhabdomyolysis that is usually characterized as fatigue, myalgia, abnormal color of urine and acute kidney damage is a potentially life-threatening syndrome accompanied by leakage of muscle fiber contents into the bloodstream.^[2]^ However, rhabdomyolysis is a rare complication in hematological malignancies,^[3,4]^ reported occasionally, and possibly caused by infection^[3,5]^ or high-dose chemotherapeutic drugs.^[6–8]^ We report a rare case of central nervous system leukemia (CNSL) complicated with rhabdomyolysis which has undefined causes.

## 2 Case report

A 20-year-old male presented with skin ecchymosis of trunk and lower limbs for 10 days. Routine inspection revealed white blood cell count (WBC) 47.7×10^9^/L in blood and 83% blasts in bone marrow. The immunophenotype of leukemia presented abnormal lymphocyte populations with CD2, CD3, CD4, CD5, CD7, CD8, CD10, CD38, Ccd3, TdT, and polymerase chain reaction (PCR) detected NOTCH EXON26 and FBXW7 EXON9 missense mutations. He was diagnosed with adult acute T-lymphocytic leukemia.

In September 2017, the patient started with hyper-Cyclophosphamide, Vincristine,Adriamycin, Dexamethasone (hyper-CVAD) regimen chemotherapy (methotrexate, pirarubicin, vincristine, and dexamethasone alternating with methotrexate and cytarabine) for 3 courses of chemotherapy. In December 2017, the patient developed intermittent distending pain in the right temporal region and blurred vision and inarticulate speech. At that time, the results of computed tomography (CT) and magnetic resonance imaging (MRI) were normal. In addition, blast-like cells were not found in bone marrow smear, and the result of cerebrospinal fluid was also negative. Still, the patient was considered as CNSL due to obvious symptoms and the patient immediately received 2 courses of chemotherapy with hyper-CVAD-B combined with PEG-ASP. Azathioprine was used for 2 weeks of maintenance chemotherapy due to inadequate supportive treatment in February 2018. During treatment, the patient developed severe depression and then citalopram hydrobromide was used to treat depression according to psychological consultant's recommendations. During the chemotherapy period, 12 lumbar punctures and intrathecal injections were performed to prevent and treat CNSL.

On the seventh day after the completion of chemotherapy, the patient complained of fever, perianal pain, and diarrhea, but he did not pay attention to it. Two days later (March 28, 2018), during our regular follow-up, he was noted to have dysuria and hematuria after catheter insertion. On admission, his axillary temperature was 36.6^o^C, his heart rate was 141 times per minute, and his blood pressure dropped to 74/54 mm Hg. Laboratory testing showed that white blood cell (WBC) and haemoglobin (HB) and platelet (PLT) decreased to 0.05×10^9^/L and 91.1 g/L and 2.8×10^9^/L, respectively; creatine kinase (CK) and creatinine which were previously normal (26 U/L and 61 μmol/L) were increased to 443 U/L and 193 μmol/L, respectively. The result of urine for occult blood test was strong positive. His coagulation function was also abnormal that prothrombin time (PT) increased to 17 seconds and activated partial thromboplastin time (APTT) increased to 70.30 s. Electrocardiogram showed sinus tachycardia. According to the past experience, vancomycin and meropenem were used to treat infection. In March 29th, he began to complain of weakness and disseminated muscle pain, and his CK increased suddenly to 3136 U/L, however, creatine kinase isoenzyme (CK-MB) was 49 U/L. At the same time, his myoglobin was noted to be markedly elevated to 813.2 ng/mL, creatinine and serum lactate dehydrogenase (LDH) were increased to 149 μmol/L and 361 U/L, respectively. Rhabdomyolysis was diagnosed and treated immediately with hydration and alkalization of urine considering severe symptoms and complication. Meropenem combined with teicoplanin was adjusted to fight infection due to renal function damage. In March 30th, the patient developed chest pain and decreased light reflex in the right eye, and the blood pressure was monitored as 70/40 mm Hg, CK and CK-MB were 2263 U/L and 59.2 U/L, respectively. Blood gas analysis suggested metabolic acidosis and repeated blood culture showed negative. He developed peripheral circulatory failure regardless of active treatment of correcting acidosis and hydration and elevated blood pressure. There was no improvement in the patient's condition and died at night despite the use of tracheal intubation, electrical defibrillation, continuous chest compression and adrenaline. The patient's father signed informed consent for publication of case details. The study was approved by the Human Ethics Committees Review Board at the First Affiliated Hospital of Guangxi Medical University, Nanning, China.

## 3 Discussion

Rhabdomyolysis is a clinical and laboratory syndrome, which refers to the changes in the integrity of the striated muscle cell membrane which leads to the release of a large number of myoglobin, CK, and small molecular substances into the peripheral blood. Rhabdomyolysis mainly manifests itself in 3 main symptoms: brown urine, muscle soreness, and weakness. There is no fixed criterion to diagnose rhabdomyolysis, but most physicians believe that CK levels are 5 or 10 times higher than normal levels in rhabdomyolysis.^[9]^ Myoglobin is not necessary for rhabdomyolysis, therefore, it is only used to assist in diagnosis.

The etiology of rhabdomyolysis is very complex including excessive exercise, muscle damage, drugs (such as statins, psychotropic drugs), alcohol, metabolic disorders, infections, and epilepsy.^[10]^ In general, the occurrence of rhabdomyolysis is caused by a variety of factors. Hence, for a particular patient, it is difficult to determine the real etiology. In this case, patient developed fever, perianal pain, and with subsequent hematuria and severe muscle pain during neutropenia after chemotherapy. The patient's serum CK and myoglobin were significantly increased, and serum creatine kinase isoenzymes-MB (CK-MB) was also increased, but the increase of CK-MB was not synchronous with CK. The disease progressed rapidly and the patient died of multiple organ failure (MODS) 3 days later despite aggressive treatment. As for the cause of rhabdomyolysis in this case, initially, because the patient received systemic chemotherapy, it was thought that side effects of chemotherapy led to rhabdomyolysis. However, these symptoms did not occur on the first chemotherapy, but on the ninth day after the fifth chemotherapy, so chemotherapy was unlikely to cause rhabdomyolysis in this case. Although hyperpyrexia can also trigger rhabdomyolysis, this usually occurs at temperatures above 42°C while the patient's maximum body temperature did not exceed 38.4°C in our record.^[11]^ Other adjoint drugs which included entecavir, citalopram hydrobromide tablets, meropenem, vancomycin, and sodium bicarbonate are currently not reported to cause rhabdomyolysis. Abnormal electrolytes can also lead to rhabdomyolysis, such as hypokalaemia, hyponatraemia, hypermatremia, hyperosmotic state, and hypophosphataemia,^[12]^ but there is no obvious abnormality in this patient. Finally, we considered that rhabdomyolysis may be caused by infection because the patient was at a state of agranulocytosis and appeared to be in septic shock. It is well known that the possibility of infection is greatly increased at a state of agranulocytosis. While, due to the low positive rate of microbial culture, the blood culture and urine culture of the patient as well as various secretions and excretion cultures were negative. Therefore, we are still not sure about the specific microorganisms of infection.

It is reported that the incidence of infectious rhabdomyolysis accounted for 5% to 19.4%, of which respiratory tract was the common site infection.^[13,14]^ The mechanisms involved in skeletal muscle damage are categorized into hypoxic, physical, chemical, or biologic.^[15]^ This patient was seriously complicated by neutropenia, septic shock, coagulant function abnormality, and MODS. Overall, the causes of rhabdomyolysis in this patient may be multidimensional, but infection is the most likely. Combined with the relevant literature, it was found that infection-induced rhabdomyolysis is rare, and the overall severity of the infection may be the basis for the pathogenesis of infection-induced rhabdomyolysis. Bone et al^[16]^ found that sepsis is usually mediated by inflammatory factors such as IL-1. Baracos et al^[17]^ revealed that the increase of prostaglandin E2 which is induced by inflammatory factors may lead to muscle fever.

Physicians should be fully aware of the possibility of rhabdomyolysis during myelosuppression after chemotherapy. Patients who develop symptoms of changes in urine, weakness, muscle soreness, or even only asymptomatic elevations in CK levels should pay attention to rhabdomyolysis and actively treated with hydration.

## Acknowledgment

We express our heartfelt thanks to the patient's parents, who agreed for this case to be shown in this paper.

## Author contributions

**Data curation:** Huiqun Li, Chengyao Wan.

**Formal analysis:** Ying Le.

**Funding acquisition:** Zhenfang Liu.

**Investigation:** Ying Le.

**Resources:** Yuan Long.

**Writing – original draft:** Ying Le.

**Writing – review & editing:** Zhenfang Liu.
